# Effect of whey protein on plasma amino acids in diabetic mice

**DOI:** 10.3892/etm.2013.1329

**Published:** 2013-10-07

**Authors:** TING HAN, DONGLIAN CAI, SHANSHAN GENG, YING WANG, HUI ZHEN, PEIYING WU

**Affiliations:** 1 Department of Clinical Nutrition, Shanghai Tenth People’s Hospital, Tongji University School of Medicine, Tongji University, Shanghai 200072, P.R. China; 2 Department of Clinical Nutrition, Changhai Hospital, The Second Military Medical University, Shanghai 200433, P.R. China; 3 Department of Clinical Nutrition, Shanghai First People’s Hospital, Shanghai Jiao Tong University School of Medicine, Shanghai 200080, P.R. China

**Keywords:** HPLC, diabetic mice, branched-chain amino acids, whey protein

## Abstract

The aim of this study was to investigate the effect of whey protein on plasma amino acid levels in a mouse model of type II diabetes, using high-performance liquid chromatography (HPLC). The composition and content of amino acids in the whey proteins were analyzed using HPLC. Type I and type II diabetic mouse models were prepared using streptozotocin (STZ) and normal mice were used as a control. The ICR mice in each group were then randomly divided into four subgroups, to which 0, 10, 20 and 40% whey protein, respectively, was administered for four weeks. Changes in the plasma amino acid levels were observed in each group. The proportions of leucine, isoleucine and valine in the whey proteins were 14.40, 5.93 and 5.32% of the total amino acids, respectively, that is, the branched-chain amino acid content was 25.65%. The levels of branched-chain amino acids increased in the plasma of the normal and model mice following the administration of whey proteins by gavage and the amino acid levels increased as the concentration of the administered protein increased. In addition, the branched-chain amino acid levels in the blood of the model mice were higher than those in the normal mice. The levels of plasma amino acids in diabetic mice increased following gavage with whey protein, which is rich in branched-chain amino acids.

## Introduction

Diabetes is a serious chronic disease threatening human health. The incidence of type II diabetes is increasing rapidly on a global scale, and the population with the disease is expected to rise from 190 million in 2000 to 360 million in 2030 (1). Of the 285 million patients with diabetes worldwide (2), the prevalence rate of adult type II diabetes is 9.7%, including a 20.4% prevalence rate in individuals >60 years of age (3). Type II diabetes is closely associated with dietary factors and cohort studies have observed that the intake of dairy products reduces the risk of type II diabetes occurring in healthy individuals (4,5). The related meta-analysis further indicated that an intake of dairy products is able to reduce the risk of the prevalence of type II diabetes, and it has been suggested that the intake of whey protein may increase the sensitivity to insulin in normal mice and improve lipid metabolism (6). In addition to impacting the metabolism of glucose and lipids, diabetes also affects the metabolism of protein and amino acids. The uptake of glucose and the insulin activity of skeletal muscles may be increased when the body’s amino acid concentration is sufficient, further promoting the synthetic metabolism of proteins (7). As such, the intake of whey protein increases the synthesis of muscle proteins (8). Amino acids are crucial materials in the activities of the body. As signaling molecules, amino acids have been shown to be important in the signal transduction of insulin secretion and glucose metabolism (9). In the present study, the amino acid levels in the whey proteins and the plasma of diabetic mice were analyzed to further investigate the mechanism of action of whey proteins on the prevention and treatment of type II diabetes.

The common methods used to detect amino acids are high-pressure liquid chromatography (HPLC), amino acid analyzers and multiple technologies involving liquid chromatography mass-spectrometry and mass spectrometry. Amino acid analyzers are simple to operate, do not require pre-column derivatization and may be used for batch testing; however, the cost is high, the detection time is relatively long and the resolution is low. The multiple technologies involving liquid chromatography-mass spectrometry and mass spectrometry are convenient and accurate; however, the cost is high. The HPLC method has numerous advantages, including a low cost, good reproducibility and high sensitivity, and, therefore, HPLC is often used as a general method for the detection of amino acids (10–12). In the present study, HPLC was used to detect the composition and content of the amino acids in whey protein and the plasma amino acid levels of mice were analyzed following gavage with whey protein. Furthermore, the mechanism of action of whey protein in the prevention of diabetes was explored. Different concentrations of whey protein were administered to ICR mouse models of type I and II diabetes. The composition and content of amino acids in whey protein were detected by HPLC, and the effect of the whey protein on plasma amino acid levels in the diabetic mice was subsequently analyzed.

## Materials and methods

### Experimental animals

Eight-week-old male ICR mice of specific pathogen free (SPF) class were purchased from the Shanghai Laboratory Animal Center, Chinese Academy of Sciences (Shanghai, China). The mice were raised in a 12-h light/dark cycle in standardized animal rooms with 40–70% relative humidity and a continuous room temperature of 18–22°C. The animal use protocol was reviewed and approved by the Institutional Animal Care and Use Committee (IACUC) of Shanghai Jiao Tong University School of Medicine (Shanghai, China).

### Model preparation

Solutions of streptozotocin (STZ; Sigma, St. Louis, MO, USA) were prepared using 0.1 mol/l citrate buffer (pH 4.2). Sodium citrate (2.1 g) was dissolved in 100 ml double distilled water to prepare solution A and 2.94 g trisodium citrate was dissolved in 100 ml double distilled water to prepare solution B. Solutions A and B were subsequently mixed with a volume ratio of 1:1.321 and the pH value was adjusted to 4.2 using 10% aqueous sodium bicarbonate, in order to prepare the desired citrate buffer. STZ (100 mg) was subsequently dissolved in 10 ml citrate buffer and filter-sterilized, prior to use. The entire preparation process was performed in an ice box.

Animals were acclimated for one week prior to the experiment. A single intraperitoneal injection of STZ (100 mg STZ solution per 1 kg body weight) was administered to the ICR mice, in order to prepare the type II diabetes model. The dose was 160 mg/kg for the preparation of the type I diabetes model (7,8). When the fasting glucose concentration was detected to be >5 mmol/l, three days later, the model was considered to be successfully prepared to meet the requirements of the experiment.

### Animal grouping and gavage

Three groups of mice were used in the experiment: Normal, a type I diabetes model and a type II diabetes model. The 32 mice in each group were then divided into four subgroups (n=8). Solutions of whey protein (0.5 ml; Australian concentrated whey protein powder with 80% purity; Davisco Foods International, Inc., Eden Prairie, MN, USA) with concentrations of 0, 10, 20 and 40% were subsequently administered to the mice in each subgroup, respectively. The aqueous solutions of whey proteins were prepared and administered at ~4:00 pm every day for four weeks; the experiment had a duration of four weeks. The eyeballs were then removed following the fasting of the mice and the blood plasma was cryopreserved.

### HPLC analysis

#### Solution preparation

Triethylamine solution was prepared by mixing 1.4 ml triethylamine with 8.6 ml acetonitrile and phenethyl isothiocyanate (PITC) acetonitrile solution was prepared by mixing one bottle (25 μl) of phenethyl isothiocyanate fat with 2 ml acetonitrile. For the mobile phase A, 15.2 g sodium acetate was dissolved in 1,850 ml water and the pH value was adjusted to 6.5 using glacial acetic acid. Following this, 140 ml acetonitrile was added and the resulting composition was mixed and filtered using a 0.45-μm membrane. For mobile phase B, 80% acetonitrile was used. In order to prepare the norleucine internal standard solution, 10 mg norleucine was dissolved and mixed in 10 ml 0.1 mol/l hydrochloric acid solution. The pure acetonitrile used in the HPLC was obtained from Concord Technology Co., Ltd. (Tianjin, China).

#### Sample treatments

Whey protein powder (2 mg) was dissolved in 1 ml Tris-HCI (pH 8.0), in order to prepare 2 mg/ml whey protein solution. To hydrolyze the whey proteins, 3.5 μl trypsin, 10 μl peptidase and 100 μl α-chymotrypsin were added to the prepared solution. The solution was mixed and warmed at 37°C in a water bath for 24 h and then stored at 4°C prior to use. To obtain a precipitate of the plasma proteins, 80 μl plasma samples were added to 100 μl perchloric acid with a concentration of 12 mol/l and centrifuged at 1,000 × g for 10 min, prior to the supernatant being transferred to a new centrifuge tube and stored at 4°C for later use.

#### Derivatization of the standard and sample solutions

Aliquots (200 μl) of the amino acid standard solutions and sample solutions were accurately measured and added to 1.5 ml Eppendorf tubes. An internal standard solution of norleucine (20 μl), 100 μl triethylamine acetonitrile and 100 μl PITC fat acetonitrile were added. After mixing and reacting at room temperature for 1 h, 400 μl n-hexane was added. The resulting mixture was then incubated with shaking for 10 min, prior to the derivative products (phenylthiocarbamoyl-amino acid solution, PTC-AA) being collected from the underlayer, filtered with a 0.45-μm needle filter and stored at 4°C for use. The amino acid analysis package was from Tianjin Bonna-Agela Technologies Co., Ltd. (Tianjin, China).

#### Chromatography conditions and the separation and determination of amino acids

The column used was a Venusil-AA amino acid analysis column measuring 4.6 mm × 250 mm × 5 μm and was obtained from Tianjin Bonna-Agela Technologies Co., Ltd. Wavelength detection was performed at 254 nm and the column temperature was 40°C. The mobile phase gradient is shown in Table I. The separation and determination of the amino acids was performed by injecting 2 μl filtrate into the HPLC column, and monitoring the chromatograms.

#### Determination of recovery rates

Three mixed branched-chain amino standards of valine (Val), leucine (Leu) and isoleucine (Ile) were accurately prepared and diluted using 0.1% hydrochloric acid solution, in order to prepare standard solutions with concentrations of 0, 50, 100, 200, 400 and 1,000 μmol/l. Val, Leu and Ile mixed standard solution (50, 200 and 1,000 μmol/l) was added to the same sample with a known concentration to determine the recovery rates.

The amino acid compositions and contents in the whey proteins and the plasma were determined by HPLC, and the amino acid levels were analyzed. In this study, norleucine was used as an internal standard to calculate the content of the other amino acids. The correction factor (f) was calculated based on the obtained peak response values of the control solution in the control substances and the internal standard substances, according to the following formula: f=(As/ms)/(Ar/mr), where As and Ar represent the peak area or peak height of the internal standard and the control, respectively, and ms and mr indicate the amount of the internal standard and the control, respectively. Following this, the samples that contained the internal standard were injected into the HPLC column and the chromatograms were recorded. According to the peak response value of the component contained in the internal standard solution being tested, the content (MI) was calculated by the flowing formula: MI=f × Ai/(As/ms), where Ai and As represent the peak area or peak height of the to-be-tested substances and the internal standard substance, respectively, and ms indicates the amount of the added internal standard. When required, the content was converted to percentage content of the labeled amount according to the dilution multiple, sample volume and labeled amount or converted into the percentage content according to the dilution multiple and the sample volume.

#### Statistical analysis

All data are presented as the mean ± standard deviation. The statistical analysis was performed using SPSS 13.0 statistical software (SPSS, Inc., Chicago, IL, USA) and significant differences were analyzed using one way analysis of variance (ANOVA). P<0.05 was considered to indicate a statistically significant difference.

## Results

### Amino acid analysis of the standard solution

**Figure 1:**
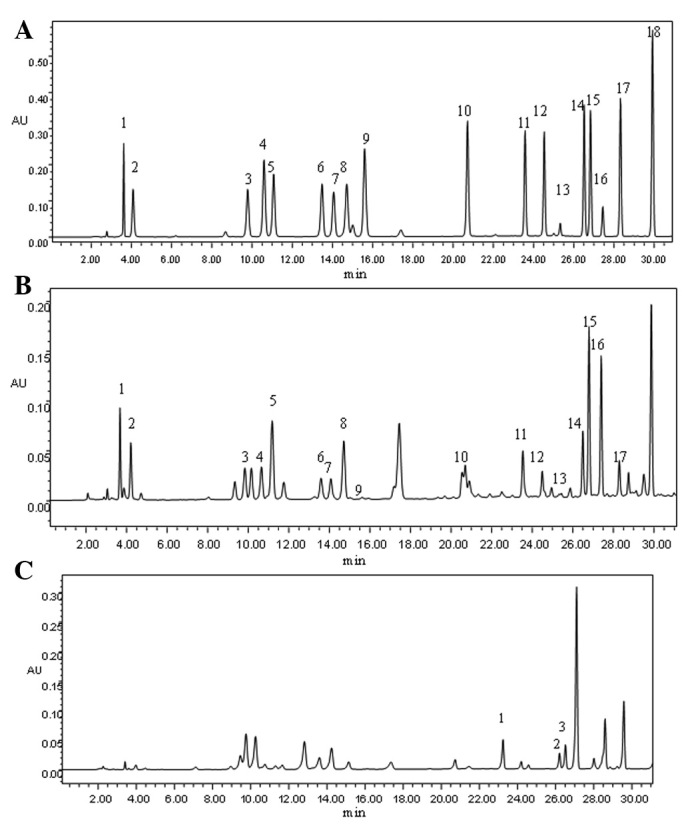
Amino acid chromatography in various samples. (A) Chromatography of the 18 amino acid standards: 1, aspartic acid (Asp); 2, glutamic acid (Glu); 3, serine (Ser); 4, glycine (Gly); 5, histidine (His); 6, arginine (Arg); 7, threonine (Thr); 8, alanine (Ala); 9, proline (Pro); 10, tyrosine (Tyr); 11, valine (Val); 12, methionine (Met); 13, cystine (Cys); 14, isoleucine (Ile); 15, leucine (Leu); 16, norleucine (Nle); 17, phenylalanine (Phe) and 18, lysine (Lys). (B) Amino acid chromatography of a 2 mg/ml solution of whey protein: 1, Asp; 2, Glu; 3, Ser; 4, Gly; 5, His; 6, Arg; 7, Thr; 8, Ala; 9, Pro; 10, Tyr; 11, Val; 12, Met; 13, Cys; 14, Ile; 15, Leu; 16, Nle; 17, Phe and 18, Lys. (C) Chromatography of plasma amino acids in the normal mouse group: 1, Val; 2, Ile; 3, Leu and 4, Internal standard (Nle).

According to the protocol stated in Materials and methods, the amino acid standards were analyzed using the appropriate column for HPLC. Standard amino acid analysis chromatogram shown in Fig. 1A, the whey protein is rich in branched chain amino acids. Analysis of the branched-chain amino acids in the present study showed that Val peaked at 23.58 min and that the peak times for Ile and Leu were similar, at 26.52 and 26.83 min, respectively. This method effectively separated each amino acid in the standard mixture. The peak for each amino acid was approximately symmetrical and the distribution curve was normal.

### Analysis of recovery rate

The recovery rates of the branched-chain amino acids were determined. Three concentrations (50, 200 and 1,000 μmol/l) were selected at which to measure the recovery rates of Val, Ile and Leu. As shown in Table II, the recovery rates of three branched-chain amino acids were all high, and the majority were >90%. The highest recovery rates were obtained when the concentration was 1,000 μmol/l.

### Analysis of whey protein

The amino acid components of the whey proteins were measured following processing with trypsin, peptidase and α-chymotrypsin. The component chromatogram and the amino acid content in the typical whey protein solution are shown in Fig. 1B. The correction factor of the internal standards in the present study was 0.0859, and the content of the amino acids was calculated using the formula shown in Materials and methods. The whey protein was rich in numerous types of amino acids, with the lysine content being measured as the highest (17.16%). The three branched-chain amino acids (Val, Ile and Leu) accounted for 25.65% of the total amino acid content (~14.40% Leu, 5.93% Ile and 5.32% Val).

### Analysis of plasma samples

**Figure 2:**
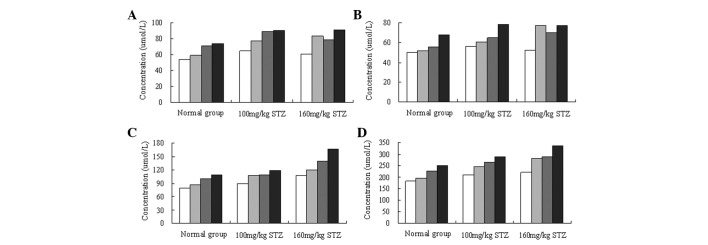
Concentrations of amino acids in the blood of mice following the intragastric administration of different concentrations of whey protein: (A) Leucine, (B) isoleucine, (C) valine and (D) branched-chain amino acids (leucine, isoleucine and valine). White bar, 0% whey protein (control group); pale gray bar, 10% whey protein; dark gray bar, 20% whey protein; black bar, 40% whey protein. 100 mg/kg streptozotocin (STZ), model of type II diabetes; 160 mg/kg STZ, model of type I diabetes.

The plasma samples were treated using the methods stated previously. The three branched-chain amino acids in the plasma samples were observed to be well separated following the amino acid chromatography (Fig. 1C). As shown in Fig. 2, the levels of branched-chain amino acids in the plasma increased in the normal and model mice. Furthermore, the increase in the levels of the branched amino acids was correlated with the concentration of the whey protein used for the gavage. In addition, the levels of branched-chain amino acids in the blood of the model mice were higher than those in the normal mice, although the differences were not observed to be significant (Table III).

## Discussion

Streptozotocin (STZ) is a reagent that is commonly used globally in the preparation of diabetic models. The intraperitoneal injection of STZ into ICR mice selectively destroys the pancreatic β cells of the mice. This method of model preparation has the advantages of low toxicity to body tissues, a high experimental animal survival rate and being simple, convenient and easy to perform. The prepared model exhibits symptoms such as high blood sugar levels, low weight, increased appetite, polydipsia and polyuria. The type II diabetic model may be prepared by the administration of STZ to 8-week-old ICR mice by intraperitoneal injection at a dose of 100 mg/kg body weight (13). In the study by Giarratana *et al*(14), it was proposed that a high-dose injection of STZ (200 mg per 1 kg body weight) was capable of inducing a type I diabetic model (14). In the present study, 100 mg STZ per 1 kg body weight was intraperitoneally injected to prepare the type II diabetic model, while 160 mg/kg was used to prepare the type I diabetic model.

A previous study has demonstrated that glucose and lipid metabolism in the blood is improved in normal mice following whey protein administration (15). As important substances for improving the protein synthesis of skeletal muscle, amino acids are likely to be widely involved in the metabolic changes (16). In order to further investigate the mechanism of action of whey protein, the composition and content of amino acids in whey protein was determined in the present study. A number of methods are commonly used to detect amino acid derivatives, including the o-phthalaldehyde (OPA) method, the PITC fat method and the 2,4-dinitrofluorobenzene method. The OPA method has been shown to elicit a fast response; however, it has been reported that the fluorescence of lysine and cystine derivatives is weak, the sensitivity is low, the glycine and lysine derivatives are unstable and the salt in the samples affects the effect of the derivatives. Furthermore, residual reagents have been shown to exert a marked impact on the column in the 2,4-dinitrofluorobenzene method, which further affects the test results. The PITC method has demonstrated the advantages of rapid analysis, high sensitivity and good repeatability (8). In the current study, in order to investigate the role of the amino acids, particularly branched-chain amino acids, present in whey protein, HPLC was used to determine the amino acid composition of the whey proteins and the amino acid levels in mice plasma. With the characteristics of rapid and highly accurate analysis, high separation efficiency, good detection performance and diverse applications, HPLC has been widely used in various fields from its inception in the 1960s and is, at present, an indispensable means of separation. In the present study, the amino acid composition and content were analyzed using HPLC. The 18 amino acid standards were well separated, and the peak of each amino acid was approximately symmetrical, with a normal distribution curve, indicating that HPLC effectively separated the amino acids.

With the characteristics of a full range of amino acids, an amino acid composition similar to the composition of amino acids essential to humans and an easy digestibility, whey protein exhibits a high bioavailability. Compared with the levels recommended by the Food and Agriculture Organization (FAO)/World Health Organization (WHO) for the amino acid content of three main proteins (whey and soy protein and casein) (17), whey protein has been shown to be rich in amino acids, particularly branched-chain amino acids, with the levels Val, Leu and Ile all being higher than those recommended by FAO/WHO. The levels of Leu and Ile in whey proteins are higher than in casein and soy proteins (18,19). In addition, the levels of histidine, threonine, methionine, cystine, phenylalanine and lysine were all higher than the recommended amounts.

In the present study, 18 amino acids were well separated from the whey protein samples, demonstrating that whey protein contains the full range of amino acids. The high levels of lysine and arginine, in particular, may stimulate the secretion of anabolic hormones to promote the growth of muscles. As a good source of sulfur-containing amino acids, such as cysteine and methionine, lysine and arginine may maintain the level of glutathione (GSH), which acts as an antioxidant. As whey protein is rich in branched-chain amino acids, the levels of branched-chain amino acids in the mouse plasma were analyzed following gavage with whey proteins, in order to further explore the role of the branched-chain amino acids. The levels of Leu, Ile and Val in the whey protein used in this study were 14.40, 5.93 and 5.32% of the total amino acid content, respectively, i.e., the branched-chain amino acid content was 25.65%. This was consistent with the results from a previous study (20).

The irreversible absorption of biological macromolecules from the plasma on the reversed phase column may lead to an increase in column pressure and reductions in column efficiency and the usage-life of the column. Therefore, it is necessary for macromolecules in the plasma to be removed prior to analysis. The method commonly used to remove proteins includes the addition on perchloric acid and centrifugation. According to preliminary experiments, perchloric acid was most efficacious at removing proteins under the conditions of the present study (21).

The levels of branched-chain amino acids in the plasma were increased following gavage with whey protein, with a higher concentration of whey protein leading to higher levels of branched-chain amino acids. In addition, the levels of branched-chain amino acids in the blood of the model mice were higher than those in the normal mice. This may have been associated with the previously reported effect of branched-chain amino acids in the improvement of insulin resistance (22).

In the present study, HPLC was demonstrated to successfully separate the amino acids in the whey proteins. The intake of whey protein increased the levels of amino acids, particularly branched-chain amino acids, in the mouse plasma. This study provided a foundation for future studies in which the mechanism of action of whey protein in the prevention of diabetes is investigated. Further studies are required to investigate the mechanism of action of the branched-chain amino acids that are abundant in the whey proteins.

## Figures and Tables

**Table I:** Gradient of the mobile phase.

Time (min)	Mobile phase A (%)	Mobile phase B (%)
0.0	100	0
2.0	100	0
15.0	90	10
25.0	70	30
33.0	55	45
33.1	0	100
38.0	0	100
38.1	100	0
45.0	100	0

**Table II:** Recovery rate from solutions containing different concentrations of Val, Leu and Ile.

	Recovery rate (%)		
	
Concentration (μmol/l)	Valine	Isoleucine	Leucine
50	92.96	84.61	94.08
200	107.56	92.77	91.98
1000	103.54	100.08	103.40

**Table III:** Plasma levels of branched-chain amino acids in the normal and model groups following whey protein gavage.

A, Normal group				

	Branched-chain amino acids (mmol/l)			
	
Whey protein (%)	Ile	Leu	Val	BCAA
0	50.01±22.31	53.27±18.84	78.99±33.64	182.26±43.78
10	51.44±25.10	58.82±17.07	87.12±36.87	197.39±59.15
20	55.19±27.40	71.19±19.32	100.26±37.21	226.65±55.27
40	68.12±29.35	74.18±24.23	108.58±40.57	250.88±64.57

B, 100 mg/kg STZ model group				

	Branched-chain amino acids (mmol/l)			
	
Whey protein (%)	Ile	Leu	Val	BCAA

0	56.03±19.00	64.19±16.14	89.13±31.54	209.35±58.74
10	60.45±24.70	77.25±27.85	107.72±33.08	245.42±67.14
20	65.12±28.00	88.61±24.94	109.51±42.75	263.23±71.07
40	78.13±34.20	90.19±28.96	119.27±52.36	287.59±106.72

C, 160 mg/kg STZ model group				

	Branched-chain amino acids (mmol/l)			
	
Whey protein (%)	Ile	Leu	Val	BCAA

0	52.10±17.05	60.86±24.51	108.03±41.25	220.98±67.04
10	77.33±25.36	83.26±30.07	120.00±43.09	280.59±76.15
20	70.15±16.34	79.05±32.64	139.89±50.07	289.09±82.47
40	77.50±28.76	91.10±36.47	166.73±60.41	335.33±92.36

100 mg/kg streptozotocin (STZ), model of type II diabetes; 160 mg/kg STZ, model of type I diabetes. Ile, isoleucine; Leu, leucine; Val, valine; BCAA, branched-chain amino acids.
